# Genome-wide association study on meningioma risk in Japan: a multicenter prospective study

**DOI:** 10.1007/s11060-024-04727-x

**Published:** 2024-07-13

**Authors:** Shuhei Yamada, Toru Umehara, Kyuto Sonehara, Noriyuki Kijima, Shuhei Kawabata, Koji Takano, Tomoki Kidani, Ryuichi Hirayama, Hideyuki Arita, Yoshiko Okita, Manabu Kinoshita, Naoki Kagawa, Toshiyuki Fujinaka, Toshiaki Fujita, Akatsuki Wakayama, Biobank Japan Project, Koichi Matsuda, Yukinori Okada, Haruhiko Kishima

**Affiliations:** 1 Department of Neurosurgery, Osaka University Graduate School of Medicine, 2-2 Yamadaoka, Suita, 565-0871 Osaka Japan; 2 Department of Neurosurgery, Hanwa Memorial Hospital, Osaka, Osaka Japan; 3 Department of Neurosurgery, Osaka International Cancer Institute, Osaka, Osaka Japan; 4 Department of Statistical Genetics, Osaka University Graduate School of Medicine, Suita, Osaka Japan; 5 Department of Genome Informatics, Graduate School of Medicine, The University of Tokyo, Tokyo, Japan; 6 Laboratory for Systems Genetics, RIKEN Center for Integrative Medical Sciences, Yokohama, Japan; 7 Department of Neurosurgery, Sakai City Medical Center, Sakai, Osaka Japan; 8 Department of Neurosurgery, National Hospital Organization Osaka National Hospital, Osaka, Osaka Japan; 9 Department of Neurosurgery, Asahikawa Medical University, Asahikawa, Hokkaido Japan; 10 Department of Neurosurgery, Osaka Neurological Institute, Toyonaka, Osaka Japan; 11 Laboratory of Clinical Genome Sequencing, Department of Computational Biology and Medical Sciences, Graduate School of Frontier Sciences, the University of Tokyo, Tokyo, Japan; 12 Laboratory of Statistical Immunology, Immunology Frontier Research Center (WPI-IFReC), Osaka University, Suita, Japan; 13 The Center for Infectious Disease Education and Research (CiDER), Osaka University, Suita, Japan

**Keywords:** Meningioma, Genome-wide association study, SNP, East Asian populations

## Abstract

**Purpose:**

Although meningiomas are the most common primary intracranial tumors, their genetic etiologies have not been fully elucidated. To date, only two genome-wide association studies (GWASs) have focused on European ancestries, despite ethnic differences in the incidence of meningiomas. The aim of this study was to conduct the first GWAS of Japanese patients with meningiomas to identify the SNPs associated with meningioma susceptibility.

**Methods:**

In this multicenter prospective case-control study, we studied 401 Japanese patients with meningioma admitted in five institutions in Japan, and 50,876 control participants of Japanese ancestry enrolled in Biobank Japan.

**Results:**

The quality control process yielded 536,319 variants and imputation resulted in 8,224,735 variants on the autosomes and 224,820 variants on the X chromosomes. This GWAS eventually revealed no genetic variants with genome-wide significance (*P* < 5 × 10 − 8) and observed no significant association in the previously reported risk variants rs11012732 and rs2686876 due to low minor allele frequency in the Japanese population.

**Conclusion:**

This is the first GWAS of meningiomas in East Asian populations and is expected to contribute to the development of GWAS research for meningiomas.

**Supplementary Information:**

The online version contains supplementary material available at 10.1007/s11060-024-04727-x.

## Introduction

Meningiomas are the most common primary intracranial tumors, accounting for at least one-third of all such lesions [1]. While the mortality rate for meningiomas is relatively lower than that for malignant glial tumors, meningiomas are associated with substantial morbidity [1, 2].

However, the etiology of meningiomas remains unknown. Exposure to ionizing radiation is a well-recognized environmental risk factor [3–5], and in males, there is a positive association between cigarette smoking and meningioma risk [6]. Evidence for inherited susceptibility to meningiomas is provided by the increased risk seen in NF2 schwannomatosis and Gorlin syndrome [7, 8]. However, these familial disorders are rare and insufficient to explain the two- to four-fold elevated risk in relatives of meningiomas [9, 10]. Furthermore, since the incidence of meningiomas varies among ethnic groups [1], the genetic predisposition in East Asian populations must be assessed separately.

A genome-wide association study (GWAS) is a powerful approach that comprehensively explores whole genomes for risk loci for various common diseases and has been utilized for risk stratification and treatment planning [11, 12]. However, the overwhelming majority of participants in the current large-scale GWAS are of European ancestry. In light of genetic heterogeneity between continental populations, it remains unclear whether these previous GWAS results can be applied to non-Europeans, including to the Japanese population [13]. In addition, the Japanese population possesses more homogeneous genetic features suitable for GWAS than other populations [14]. The expansion of single ethnic GWAS, like that for the Japanese population, has the potential to reveal novel susceptibility loci not only for ethnicity-specific loci but also for common loci across different ethnicities, even if its large-scale GWAS have been conducted globally [15]. It is conceivable that the same can be true of GWAS in meningioma. Two susceptibility single nucleotide polymorphisms (SNPs), such as rs11012732 and rs2686876, have been reported in two GWAS reports for meningioma; however, those studies mainly focused on European ancestries [16, 17].

Given this background, we performed the first GWAS of Japanese patients with meningiomas to identify SNPs associated with meningioma susceptibility.

## Methods

### Ethics

Appropriate approval was obtained from the local institutional review board (approval number 846-3), before initiating the study. Each patient was fully informed of the study and provided written informed consent prior to participation.

### Study design and patient selection

Patients with pathologically diagnosed or radiologically suspected meningiomas were selected from five institutions (Osaka University Hospital, Osaka, Japan; Hanwa Memorial Hospital, Osaka, Japan; Osaka International Cancer Institute, Osaka, Japan; Osaka Neurological Institute, Osaka, Japan; and Osaka National Hospital, Osaka, Japan) between September 2019 and April 2021. Patients under 15 years or who did not consent to the study were excluded. Control participants were enrolled by BioBank Japan [18, 19].

### Genotype quality control

A total of 426 patients with meningioma and 54,406 control participants were genotyped using an Infinium Asian Screening Array chip (Illumina, Inc., San Diego, CA, USA). This genotyping array was built using an East Asian reference panel, including WGS, which enabled efficient genotyping of East Asian populations.

We applied stringent quality control (QC) filters to both samples and variants as described elsewhere [20]. Briefly, for sample QC, we excluded individuals with a low call rate (< 0.98) and outliers from the cluster of East Asian populations in PCA that was conducted together with the samples of HapMap Phase II. For a more stringent quality control for population stratification, we additionally excluded outliers from the Hondo cluster based on PCA [21]. For variant QC, we excluded SNPs (i) with a low call rate (< 0.99); (ii) with low minor allele counts (< 5); (iii) with Hardy–Weinberg equilibrium test *P*-value < 1.0 × 10^−10^; and (iv) with more than 0.05 of allele frequency difference when compared with the representative reference panels of Japanese ancestry (i.e., the imputation reference panel described below [22] and the allele frequency panel of Tohoku Medical Megabank Project [23].

As a result of genotype QC, 25 cases and 3,530 individuals from controls were excluded from further analyses. Finally, the sample size available for the GWAS analyses included 401 cases and 50,876 controls.

### GWAS imputation

We used the SHAPEIT4 software [24] for haplotype phasing and Minimac4 software [25] for genotype imputation. As an imputation reference, we used the combined reference panel of 1KG phase 3v5a (*n* = 2,504) and Japanese whole-genome sequencing data (*n* = 1,037) [21]. We used imputed variants with an imputation score of *Rsq* ≥ 0.7 and minor allele frequency (MAF) ≥ 0.5%. We additionally queried MAFs of important SNPs via dbSNP at the National Center for Biotechnology Information (www.ncbi.nlm.nih.gov/projects/SNP/) [26].

### Tumor location

We divided tumor locations into skull base, non-skull base, and extra-cranial, as in a previous report from our institution [27]. Lesions in the olfactory groove, planum sphenoidale, cavernous sinus, sphenoid wing, clinoidal portion, tuberculum sellae, clivus, and petrous bone were classified as skull base lesions. Furthermore, we included the optic nerve sheath as a skull base lesion.

### Statistical analysis

We performed a case-control analysis with imputed genotype dosage as an explanatory variable, using the Scalable and Accurate Implementation of Generalized mixed model [28], considering unbalanced case-control ratios and sample relatedness. Power calculations were completed using CaTS Power Calculator software [29]. In these calculations, the prevalence of meningiomas was set to 2.5% [30] and the odds ratio (OR) for each risk allele of the tested genetic variants was approximately 1.6, as estimated in previous studies [16, 17]. Subsequently, stratified GWAS analyses for meningiomas were conducted according to tumor location and sex, focusing on their varying molecular subgroup between skull base and non-skull base [31–33] and on their gender differences in the prevalence [34, 35]. To correct for population structure bias, we included the top five components obtained from PCA in the covariates of the regression analysis. We also included sex as a covariate in the regression analysis when analyzing variants on the X chromosome. SNPs with *p*-values < 5 × 10^−8^ are considered genome-wide significant [36].

## Results

We studied 401 meningioma patients of Japanese ancestry and 50,876 control participants of Japanese ancestry. The clinical characteristics of the patients are presented in Table 1. Consistent with the Report of the Brain Tumor Registry of Japan (2005–2008) [34], the incidence rate of meningiomas in women was more than twice that in men in our dataset. The QC process yielded 536,319 variants remaining, and imputation resulted in 8,224,735 variants on autosomes and 224,820 variants on X chromosomes. With the sample size (401 cases and 50,876 controls), the statical power by the MAFs to achieve genome-wide significant SNP associated with meningiomas at *p*-values < 5 × 10^−8^ was shown in Supplementary Fig. 1. The statical powers are sufficient (> 80%) if the MAF is set to 30% or more, which is equivalent to that of the reported susceptibility of SNPs in previous reports [16, 17], suggesting that the sample size in our study is appropriate.

**Table 1:** Clinical characteristics of the meningioma cases and control participants

*n* (%) / mean ± SD	Meningioma cases (*n* = 401)	Control participants (*n* = 50,876)
Age (y)	68.3 ± 12.5	65.1 ± 13.7
Female	302 (75.3)	24,409 (48.0)
Multiple	31 (7.7)	N/A
Tumor location		
Non skull-base	244 (60.8)	N/A
Skull-base	151 (37.7)	N/A
Extracranial	6 (1.5)	N/A

**Fig. 1:**
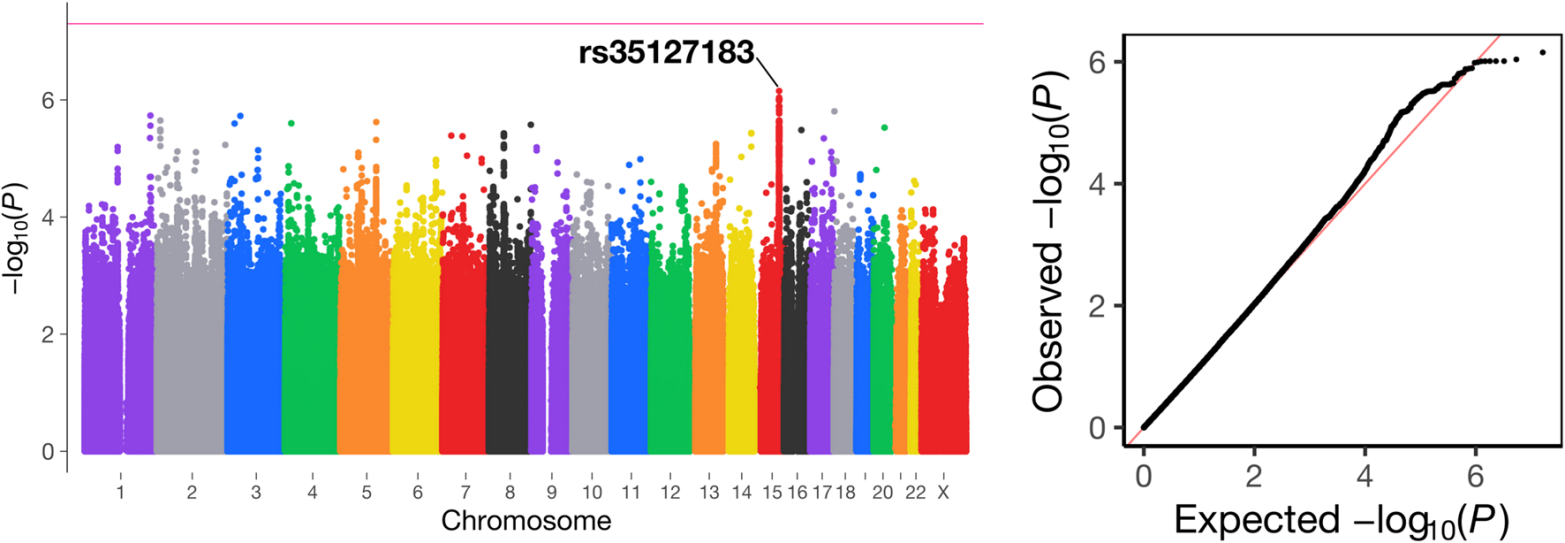
Manhattan plot of a genome-wide association study of meningiomas in a Japanese population. The horizontal red line indicates the genome-wide significance threshold (*P* = 5.0 × 10 − 8). Q-Q plot of the observed *P*-value (-log10P) for the meningioma cases and the controls (λ = 1.02)

**Fig. 2:**
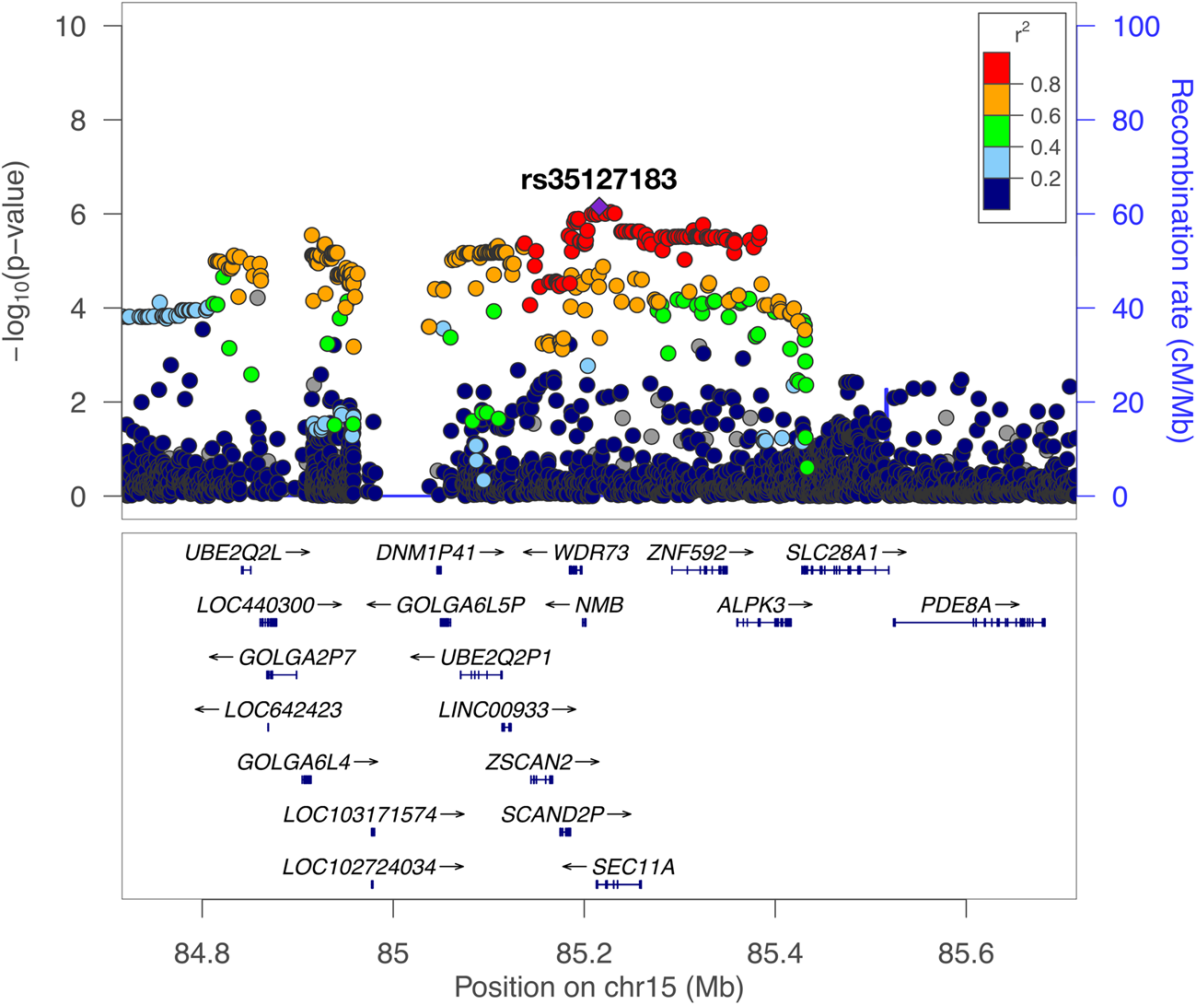
A regional plot of the chromosome 15q The lead variant (rs35127183) is colored purple, and all other variants are colored based on linkage disequilibrium (LD) with the lead variant, as in the legend. The LD statistics for r2 were calculated using the East Asian reference panel of the 1000 Genomes Project Phase 3 version 5

This GWAS in the overall dataset eventually revealed no genetic variants with genome-wide significance (*P* < 5 × 10^−8^) (Fig. 1). The strongest association was provided by SNP rs35127183 on 15q25, where the A allele was associated with the increased risk (Odds ratio [OR], 1.63; 95% confidence interval [CI], 1.34–1.99; *P* = 7.0 × 10^−7^; Non-risk allele, G). The risk allele frequency was 0.17 in the cases and 0.11 in the controls. The SNP rs35127183 is an intron variant of the *SEC11A* gene (Fig. 2).

Among the reported susceptibility SNPs for meningiomas, rs11012732 [16] was covered using the Infinium Asian Screening Array chip. The MAF was 0.87% in the case group and 0.54% in the control group. Alternatively, rs2686876 [17] was not included in the chip; however, its imputation quality was high (imputation score = 0.99). The MAF was 0.62% in cases and 1.3% in controls. A significant association was not observed for either rs11012732 (*P* = 0.26) or rs2686876 (*P* = 0.21) and thus was incapable of validating previously reported risk variants.

In the stratified GWAS analyses according to tumor location and sex, patients with extracranial meningiomas and all male individuals were excluded because of their small sample sizes, as shown in Table 1. The tumor location-stratified GWAS on both skull base (151 for cases and 50,876 for controls) and non-skull base meningiomas (244 for cases and 50,876 for controls) also revealed no genetic variants with genome-wide significance (*P* < 5 × 10^−8^) (Supplementary Fig. 2 and Supplementary Fig. 3). In the sex-stratified GWAS on female (302 for cases and 24,409 for controls), we identified one variant associated with the risk of meningioma that satisfied the genome-wide significance (*P* = 4.7 × 10^−8^) (Supplementary Fig. 4). The variant was SNP rs141887933 on 2p25, where the T allele was associated with the increased risk (OR, 3.98; 95% CI, 2.40–6.61; Non-risk allele, G), the MAF was 4.1% in cases and 1.1% in controls.

## Discussion

This GWAS on meningioma was intended to gain new insights into the genetic loci associated with the risk of meningioma in Japan, as the Japanese population possesses homogeneous genetic features. The power analysis suggested that a sufficient sample size had been secured to detect SNPs associated with meningiomas in East Asian populations, assuming the susceptibility of SNP with a MAF of approximately 30% and an odds ratio of approximately 1.6, which are like those in previous GWAS reports; however, it was unable to identify novel loci associated with meningioma. The lead variant in the overall dataset, although without genome-wide significance, was rs35127183, an intronic *SEC11A* variant of unknown clinical significance. The *SEC11A* gene encodes the signal peptidase complex 18, which contributes to malignant progression via promotion of transforming growth factor alpha secretion in gastric cancer [37]. The rs35127183 is relatively common in Europeans (MAF > 30%) and East Asians (10%> MAF > 5%); therefore, the MAF disjunction is less remarkable between Europeans and East Asians [26]. Considering the absence of relevant data on rs35127183 from previous large-scale GWAS, it is unlikely that this intron variant is a universal biomarker for meningiomas.

To verify the results of previous studies, we attempted to investigate whether rs11012732 and rs2686876 are correlated with meningiomas [16, 17]. However, it was difficult to determine their relevance, probably because of the lack of statistical power due to their low MAFs in the Japanese population. The rs11012732 and rs2686876 are rarely prevalent in East Asian populations (MAF < 1%), whereas they are common genetic variants in European populations (MAF > 30%). Based on the MAF heterogeneity of key variants, divergent results between previous GWAS and this study were expected. To compensate for the lower MAFs and achieve adequate statistical power in the Japanese population, the study requires a much larger sample size in a high proportion of cases, which are areas for improvement. The results of this study are far from conclusive that rs11012732 and rs2686876 are independent of meningioma exclusively in the Japanese population.

Stratified GWAS analyses for meningiomas according to tumor location and sex were additionally conducted in this study since meningiomas are more common in women [34, 35] and the molecular subgroups, having biallelic loss of the neurofibromatosis 2 gene at the top of list, vary between skull base and non-skull base [31–33]. Especially in a sex-stratified GWAS of female, rs141887933 on 2p25 was significantly associated with the risk of meningioma. rs141887933, which is also an intronic *GREB1* variant of unknown clinical significance, is rarely prevalent in European or East Asian populations (MAF < 1%). Although this is the sole variant in this study that achieved genome-wide significance, this statistical data interpretation requires careful consideration considering the low-frequency variant detection and potential multiple comparisons in the sex-stratified GWAS.

To the best of our knowledge, this is the first GWAS of meningioma in East Asian populations that utilized 401 patients with meningioma and 50,876 control participants in the Japanese population. We believe that data reporting will provide a reasonable base and contribute to the development of meningioma research in the future.

## Conclusions

We conducted a GWAS of meningiomas in the Japanese population; however, no significant genetic variants were identified, except in the sex-stratified GWAS of females. This study also had difficulty validating previously reported risk variants associated with meningiomas, possibly because of heterogeneous MAFs in ancestrally diverse populations.

### Supplementary Information

Below is the link to the electronic supplementary material.

Supplementary file1 (TIF 12018 KB)

Supplementary file2 (TIF 12018 KB)

Supplementary file3 (TIF 12018 KB)

Supplementary file4 (DOCX 15 KB)

## Data Availability

The anonymized data that support the findings of this study are available from the corresponding author upon reasonable request.

## References

[1] Ostrom QT, Patil N, Cioffi G et al (2020) CBTRUS Statistical Report: primary brain and other Central Nervous System tumors diagnosed in the United States in 2013–2017. Neuro Oncol 22:iv1–iv96. 10.1093/neuonc/noaa200 33123732 10.1093/neuonc/noaa200 PMC7596247

[2] Magill ST, Dalle Ore CL, Diaz MA et al (2019) Surgical outcomes after reoperation for recurrent non–skull base meningiomas. J Neurosurg 131:1179–1187. 10.3171/2018.6.JNS18118 30544357 10.3171/2018.6.JNS18118

[3] Wiemels J, Wrensch M, Claus EB (2010) Epidemiology and etiology of meningioma. J Neurooncol 99:307–314. 10.1007/s11060-010-0386-3 20821343 10.1007/s11060-010-0386-3 PMC2945461

[4] Ogasawara C, Philbrick BD, Adamson DC (2021) Meningioma: a review of epidemiology, pathology, diagnosis, treatment, and future directions. Biomedicines 9. 10.3390/biomedicines9030319 10.3390/biomedicines9030319 PMC8004084 33801089

[5] Braganza MZ, Kitahara CM, Berrington De González A et al (2012) Ionizing radiation and the risk of brain and central nervous system tumors: a systematic review. Neuro Oncol 14:1316–1324. 10.1093/neuonc/nos208 22952197 10.1093/neuonc/nos208 PMC3480263

[6] Chao H, Cheng Y, Shan J et al (2021) A meta-analysis of active smoking and risk of meningioma. Tob Induc Dis 19:14–17. 10.18332/TID/133704 10.18332/TID/133704 PMC8106389 33994906

[7] Kerr K, Qualmann K, Esquenazi Y et al (2018) Familial syndromes involving meningiomas provide mechanistic insight into sporadic disease. Neurosurgery 83:1107–1118. 10.1093/neuros/nyy121 29660026 10.1093/neuros/nyy121 PMC6235681

[8] Asthagiri AR, Parry DM, Butman JA et al (2009) Neurofibromatosis type 2. Lancet 373:1974–1986. 10.1016/S0140-6736(09)60259-2 19476995 10.1016/S0140-6736(09)60259-2 PMC4748851

[9] Malmer B, Henriksson R, Grönberg H (2003) Familial brain tumours - Genetics or environment? A nationwide cohort study of cancer risk in spouses and first-degree relatives of brain tumour patients. Int J Cancer 106:260–263. 10.1002/ijc.11213 12800203 10.1002/ijc.11213

[10] Claus EB, Calvocoressi L, Bondy ML et al (2011) Family and personal medical history and risk of meningioma: clinical article. J Neurosurg 115:1072–1077. 10.3171/2011.6.JNS11129 21780859 10.3171/2011.6.JNS11129 PMC3241000

[11] Tam V, Patel N, Turcotte M et al (2019) Benefits and limitations of genome-wide association studies. Nat Rev Genet 20:467–484. 10.1038/s41576-019-0127-1 31068683 10.1038/s41576-019-0127-1

[12] Khera AV, Chaffin M, Aragam KG et al (2018) Genome-wide polygenic scores for common diseases identify individuals with risk equivalent to monogenic mutations. Nat Genet 50:1219–1224. 10.1038/s41588-018-0183-z 30104762 10.1038/s41588-018-0183-z PMC6128408

[13] Martin AR, Kanai M, Kamatani Y et al (2019) Clinical use of current polygenic risk scores may exacerbate health disparities. Nat Genet 51:584–591. 10.1038/s41588-019-0379-x 30926966 10.1038/s41588-019-0379-x PMC6563838

[14] Haga H, Yamada R, Ohnishi Y et al (2002) Gene-based SNP discovery as part of the japanese Millennium Genome Project: identification of 190 562 genetic variations in the human genome. J Hum Genet 47:605–610. 10.1007/s100380200092 12436197 10.1007/s100380200092

[15] Imamura M, Takahashi A, Yamauchi T et al (2016) Genome-wide association studies in the Japanese population identify seven novel loci for type 2 diabetes. Nat Commun 7. 10.1038/ncomms10531 10.1038/ncomms10531 PMC4738362 26818947

[16] Dobbins SE, Broderick P, Melin B et al (2011) Common variation at 10p12.31 near MLLT10 influences meningioma risk. Nat Genet 43:825–827. 10.1038/ng.879 21804547 10.1038/ng.879 PMC5053355

[17] Claus EB, Cornish AJ, Broderick P et al (2018) Genome-wide association analysis identifies a meningioma risk locus at 11p15.5. Neuro Oncol 20:1485–1493. 10.1093/neuonc/noy077 29762745 10.1093/neuonc/noy077 PMC6176799

[18] Nagai A, Hirata M, Kamatani Y et al (2017) Overview of the BioBank Japan Project: study design and profile. J Epidemiol 27:S2–S8. 10.1016/j.je.2016.12.005 28189464 10.1016/j.je.2016.12.005 PMC5350590

[19] Hirata M, Kamatani Y, Nagai A et al (2017) Cross-sectional analysis of BioBank Japan clinical data: a large cohort of 200,000 patients with 47 common diseases. J Epidemiol 27:S9–S21. 10.1016/j.je.2016.12.003 28190657 10.1016/j.je.2016.12.003 PMC5363792

[20] Sonehara K, Kimura Y, Nakano Y et al (2022) A common deletion at BAK1 reduces enhancer activity and confers risk of intracranial germ cell tumors. Nat Commun 13. 10.1038/s41467-022-32005-9 10.1038/s41467-022-32005-9 PMC9346128 35918310

[21] Okada Y, Momozawa Y, Sakaue S et al (2018) Deep whole-genome sequencing reveals recent selection signatures linked to evolution and disease risk of Japanese. Nat Commun 9:1–10. 10.1038/s41467-018-03274-0 29691385 10.1038/s41467-018-03274-0 PMC5915442

[22] Akiyama M, Ishigaki K, Sakaue S et al (2019) Characterizing rare and low-frequency height-associated variants in the Japanese population. Nat Commun 10. 10.1038/s41467-019-12276-5 10.1038/s41467-019-12276-5 PMC6764965 31562340

[23] Tadaka S, Hishinuma E, Komaki S et al (2021) jMorp updates in 2020: large enhancement of multi-omics data resources on the general Japanese population. Nucleic Acids Res 49:D536–D544. 10.1093/nar/gkaa1034 33179747 10.1093/nar/gkaa1034 PMC7779038

[24] Delaneau O, Marchini J, Zagury JF (2012) A linear complexity phasing method for thousands of genomes. Nat Methods 9:179–181. 10.1038/nmeth.1785 10.1038/nmeth.1785 22138821

[25] Das S, Forer L, Schönherr S et al (2016) Next-generation genotype imputation service and methods. Nat Genet 48:1284–1287. 10.1038/ng.3656 27571263 10.1038/ng.3656 PMC5157836

[26] Bhagwat M (2010) Searching NCBI’s dbSNP database. Curr Protoc Bioinforma. 10.1002/0471250953.bi0119s32 10.1002/0471250953.bi0119s32 PMC3078622 21154707

[27] Yamada S, Kinoshita M, Nakagawa T et al (2021) The impact of 5-Year Tumor Doubling Time to predict the subsequent long-term natural history of asymptomatic meningiomas. World Neurosurg 151:e943–e949. 10.1016/j.wneu.2021.05.023 34020064 10.1016/j.wneu.2021.05.023

[28] Zhou W, Nielsen JB, Fritsche LG et al (2018) Efficiently controlling for case-control imbalance and sample relatedness in large-scale genetic association studies. Nat Genet 50:1335–1341. 10.1038/s41588-018-0184-y 30104761 10.1038/s41588-018-0184-y PMC6119127

[29] Skol AD, Scott LJ, Abecasis GR, Boehnke M (2006) Joint analysis is more efficient than replication-based analysis for two-stage genome-wide association studies. Nat Genet 38:209–213. 10.1038/ng1706 16415888 10.1038/ng1706

[30] Bos D, Poels MMF, Adams HHH et al (2016) Prevalence, Clinical Management, and natural course of incidental findings on brain MR images: the Population-based Rotterdam scan study. Radiology 281:507–515. 10.1148/radiol.2016160218 27337027 10.1148/radiol.2016160218

[31] Clark VE, Erson-Omay EZ, Serin A et al (2013) Genomic analysis of non-NF2 meningiomas reveals mutations in TRAF7, KLF4, AKT1, and SMO. Science 339:1077–1080. 10.1126/science.1233009 23348505 10.1126/science.1233009 PMC4808587

[32] Brastianos PK, Horowitz PM, Santagata S et al (2013) Genomic sequencing of meningiomas identifies oncogenic SMO and AKT1 mutations. Nat Genet 45:285–289. 10.1038/ng.2526 23334667 10.1038/ng.2526 PMC3739288

[33] Okano A, Miyawaki S, Hongo H et al (2021) Associations of pathological diagnosis and genetic abnormalities in meningiomas with the embryological origins of the meninges. Sci Rep 11:1–13. 10.1038/s41598-021-86298-9 33772057 10.1038/s41598-021-86298-9 PMC7998008

[34] (2017) Brain Tumor Registry of Japan (2005–2008). Neurol Med Chir (Tokyo) 57:9–102. 10.2176/nmc.sup.2017-0001 10.2176/nmc.sup.2017-0001 PMC6760096 28420810

[35] Matsumoto F, Takeshima H, Yamashita S et al (2021) Epidemiologic study of primary brain tumors in Miyazaki prefecture: a regional 10-year survey in southern Japan. Neurol Med Chir (Tokyo) 61:492–498. 10.2176/nmc.oa.2020-0438 34148943 10.2176/nmc.oa.2020-0438 PMC8365235

[36] Pe’er I, Yelensky R, Altshuler D, Daly MJ, (2008) Estimation of the multiple testing burden for genomewide association studies of nearly all common variants. Genet Epidemiol 32:381–385. 10.1002/gepi.20303 18348202 10.1002/gepi.20303

[37] Oue N, Naito Y, Hayashi T et al (2014) Signal peptidase complex 18, encoded by SEC11A, contributes to progression via TGF-α secretion in gastric cancer. Oncogene 33:3918–3926. 10.1038/onc.2013.364 23995782 10.1038/onc.2013.364

