# Supplementary figures and images for: Genome-wide association study on meningioma risk in Japan: a multicenter prospective study
Source: J Neurooncol. 2024 Jul 13;169(2):281–6. doi: 10.1007/s11060-024-04727-x (PMC11341637; doi:10.1007/s11060-024-04727-x)

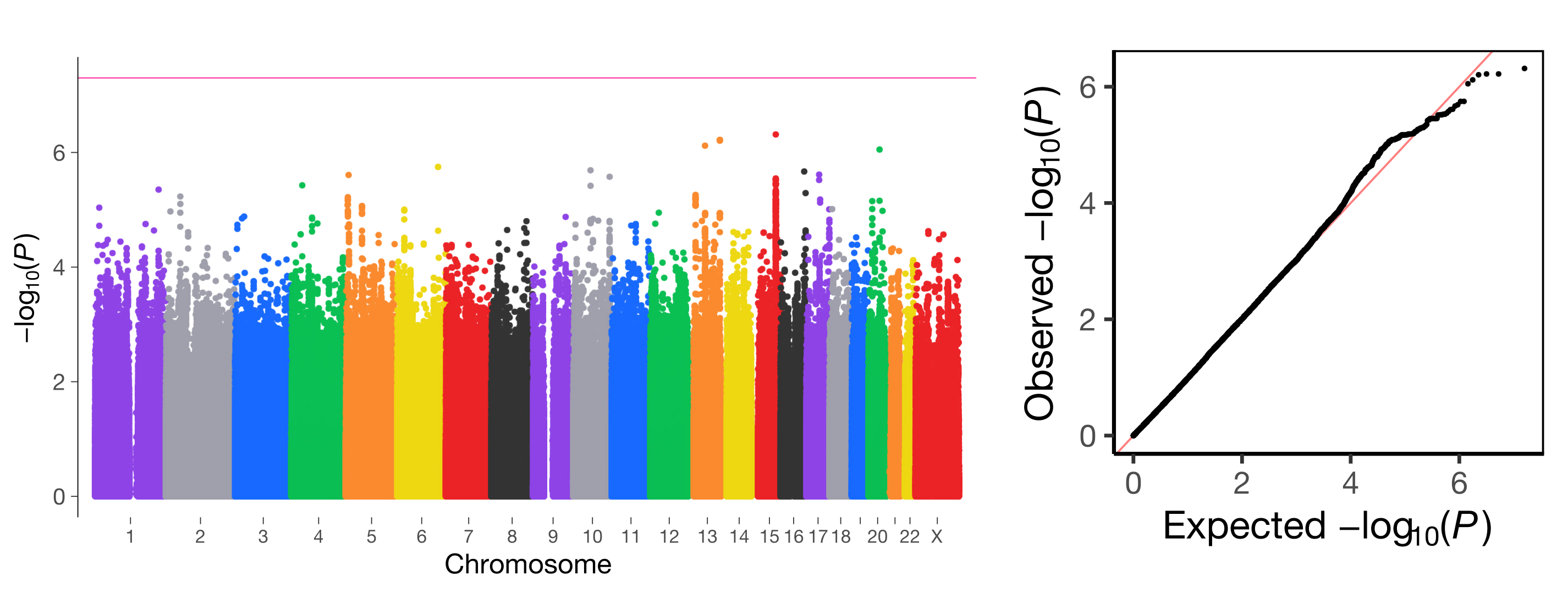

Supplement: Supplementary file 1 — Supplementary file1 (TIF 12018 KB) [file 11060_2024_4727_MOESM1_ESM.tif]

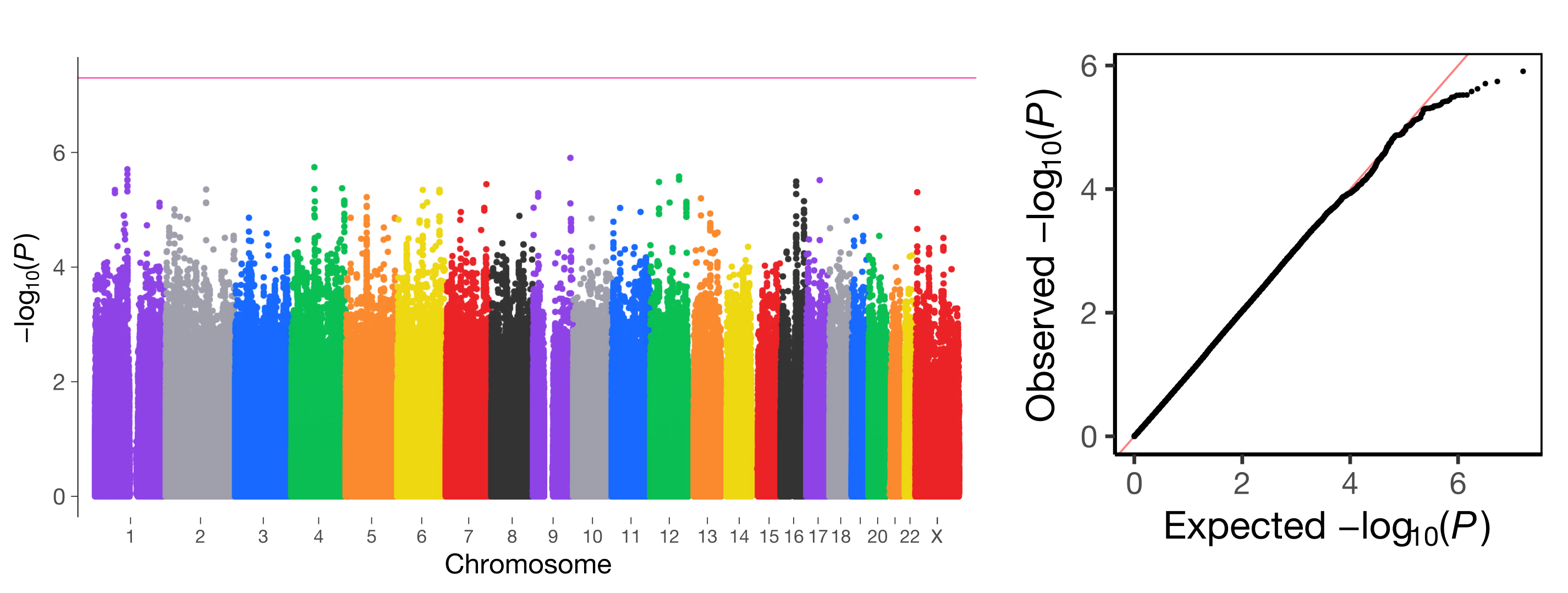

Supplement: Supplementary file 2 — Supplementary file2 (TIF 12018 KB) [file 11060_2024_4727_MOESM2_ESM.tif]

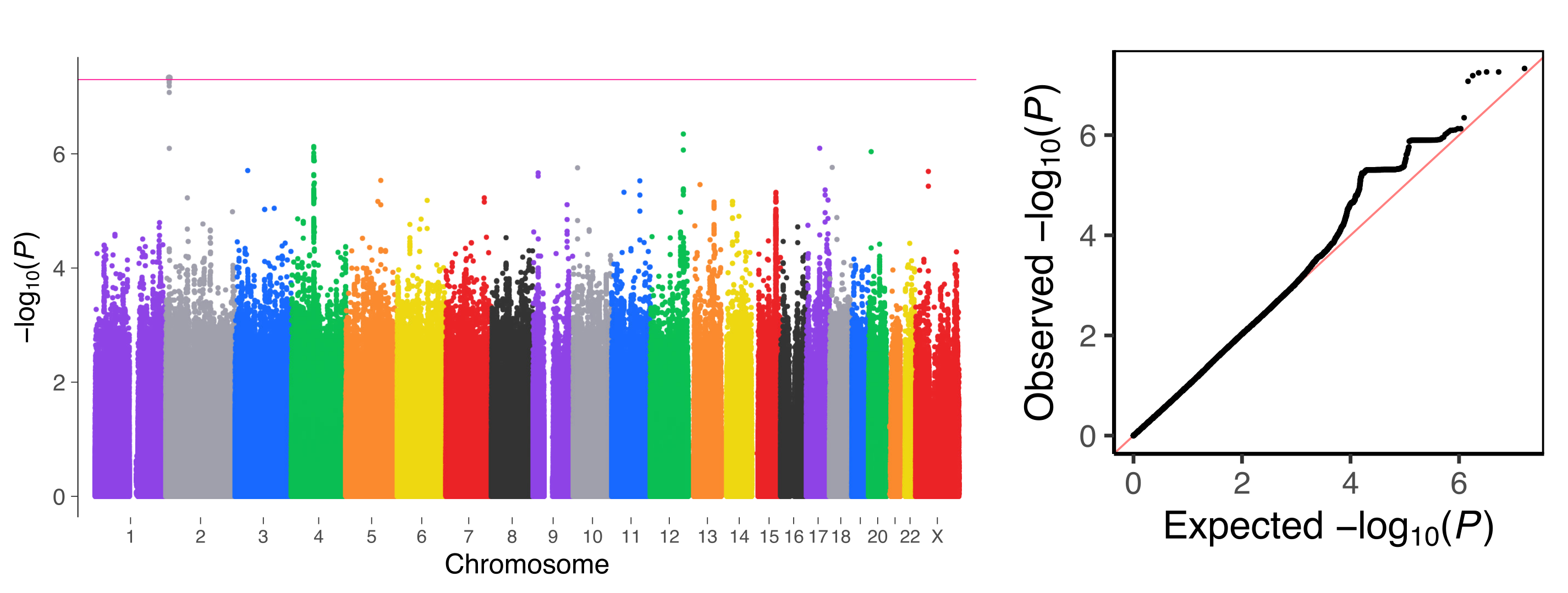

Supplement: Supplementary file 3 — Supplementary file3 (TIF 12018 KB) [file 11060_2024_4727_MOESM3_ESM.tif]
