# Supplementary material for: Genome-wide association study on meningioma risk in Japan: a multicenter prospective study
Source: J Neurooncol. 2024 Jul 13;169(2):281–6. doi: 10.1007/s11060-024-04727-x (PMC11341637; doi:10.1007/s11060-024-04727-x)
Supplement: Supplementary file 4 — Supplementary file4 (DOCX 15 KB) [file 11060_2024_4727_MOESM4_ESM.docx]

**Supplementary Figure Legends**

**Supplementary Figure 1**

(A) A Manhattan plot of the genome-wide association study of skull base meningiomas in the Japanese population

The horizontal red line indicates the genome-wide significance threshold (*P* = 5.0×10^-8^).

(B) Q-Q plot of the observed *P*-value (-log_10_P) for the meningioma cases and the controls (λ = 0.96)

**Supplementary Figure 2**

(A) A Manhattan plot of the genome-wide association study of non-skull base meningiomas in the Japanese population

The horizontal red line indicates the genome-wide significance threshold (*P* = 5.0×10^-8^).

(B) Q-Q plot of the observed *P*-value (-log_10_P) for the meningioma cases and the controls (λ = 0.98)

**Supplementary Figure 3**

(A) A Manhattan plot of the genome-wide association study of females with meningiomas in the Japanese population

The horizontal red line indicates the genome-wide significance threshold (*P* = 5.0×10^-8^).

(B) Q-Q plot of the observed *P*-value (-log_10_P) for the meningioma cases and the controls (λ = 0.97)
